# Integrated omics analysis of PGPR and AMF effects on soil microbiota and root metabolites in *Isatis indigotica*

**DOI:** 10.3389/frmbi.2025.1709335

**Published:** 2025-11-17

**Authors:** Shuai Yang, Ting Yuan, Tingting Duan, Huimin Zhu, Xiaoxia Zhang, Haihong Zhang, Junjie Jiang, Jun Yang, Lichuan Hu, Yu Yu, Lijun Zhang, Zhaodi Yuan

**Affiliations:** 1 School of Biology and Pharmacy, Mianyang Normal University, Mianyang, Sichuan, China; 2 Grain and Oil Research Institute, Tongren Academy of Agricultural Sciences, Tongren, Guizhou, China; 3 School of Life Sciences, Guizhou University, Guiyang, Guizhou, China

**Keywords:** *Isatis indigotica*, plant growth-promoting rhizobacteria (PGPR), arbuscular mycorrhizal fungi (AMF), rhizosphere microbiome, root metabolomics, biofertilizers

## Abstract

**Introduction:**

The intensive use of chemical fertilizers and pesticides in modern agriculture has led to severe soil degradation and environmental pollution, which threatens the long-term production of crops. Plant growth-promoting rhizobacteria (PGPR) and arbuscular mycorrhizal fungi (AMF) are promising biofertilizers which can boost plant growth and improve soil quality. However, the combined effects of these factors on medicinal plants such as *Isatis indigotica* remain unclear.

**Methods:**

This study isolated and identified six plant growth-promoting rhizobacteria (PGPR) strains (*Acinetobacter sp*. and *Bacillus albus*) from the rhizosphere of *Isatis indigotica*. A pot experiment was conducted with control, PGPR inoculation and AMF+PGPR co-inoculation treatments to assess the effects of these treatments on the growth of *Isatis indigotica* and its soil physicochemical properties. High-throughput sequencing was used to analyse the structure of the rhizosphere microbial community, while non-targeted metabolomics was employed to profile root metabolites. Finally, a redundancy analysis (RDA) was performed to reveal the correlations between the key microbial taxa and the differential metabolites.

**Results:**

All six of the isolated PGPR strains exhibited multiple capacities that promote plant growth. The pot experiment demonstrated that both PGPR inoculation and AMF+PGPR co-inoculation significantly increased the height and root length of *Isatis indigotica* compared to the control, while also enhancing the soil’s SOC, TN and AP content. Analysis of the microbial community revealed that the inoculation treatments enriched the rhizosphere microbiome with beneficial taxa such as *Proteobacteria* and *Ascomycota*. Metabolomic analysis revealed that inoculation treatments significantly increased the concentrations of key bioactive compounds, such as flavonoids, lipids and amino acids. Furthermore, the RDA revealed a strong correlation between the accumulation of various root metabolites (e.g., benzenesulfonic acids, carbohydrates and fatty acids) and dominant microbial genera (e.g., *Acinetobacter*, *Paenibacillus* and *Botryotrichum*).

**Conclusions:**

PGPR and AMF improve the uptake of nutrients and the synthesis of secondary metabolites in *Isatis indigotica* by altering the structure of the rhizosphere microbiome and root metabolomes. These findings support the use of PGPR and AMF as biofertilizers for sustainably cultivating medicinal plants.

## 1 Introduction

The intensive use of chemical fertilizers and pesticides in modern agriculture has led to severe soil degradation and environmental pollution, which poses a significant threat to the sustainability of ecosystems (Agarwal et al., 2018; Zulfiqar et al., 2019; Atieno et al., 2020; Vejan et al., 2021). Excessive nitrogen and phosphorus inputs can disrupt soil microbial communities, reduce the content of organic matter and accelerate soil acidification. Ultimately, this diminishes long-term agricultural productivity (Freedman et al., 2015; Tian et al., 2020; Wang et al., 2022; Qin et al., 2024). The overuse of synthetic pesticides has been shown to have a negative impact on beneficial microorganisms, resulting in ecosystem degradation, decreased productivity and poor crop quality (Liu et al., 2015; Banerjee and van der Heijden, 2023; Khangura et al., 2023). Therefore, this unsustainable paradigm requires the urgent adoption of microbial-based alternatives that can maintain crop productivity while restoring soil health. This is a challenge that PGPR-AMF consortia are well placed to address, given their dual roles in nutrient cycling and pathogen suppression (Fasusi et al., 2023).

Plant growth-promoting rhizobacteria (PGPR) can colonize and proliferate within the plant microbiome in the rhizosphere environment (Lugtenberg and Kamilova, 2009; Compant et al., 2019). Furthermore, microorganisms in the rhizosphere communicate with plant roots, influencing their function and playing a significant role in plant health, nutrition and yield (Habibi et al., 2014). PGPRs can be divided into two types according to their modes of action: those with a direct effect and those with an indirect effect. PGPRs can directly promote plant growth by secreting plant hormones such as auxins (e.g. indole-3-acetic acid, IAA), or by enhancing nutrient availability through nitrogen fixation and phosphate solubilization (Ahmad et al., 2008; Habibi et al., 2014). They produced siderophores and ACC-deaminase enzymes, which suppress infection by pathogenic bacteria and fungi, and act as biocontrol agents, indirectly promote plants growth (Barea et al., 2005; de los Santos-Villalobos et al., 2012; Santoyo et al., 2021). The soil’s physical and chemical properties were also improved, which could potentially reduce the need for chemical fertilizers (Orozco-Mosqueda et al., 2018; Santoyo et al., 2021). Furthermore, PGPR can significantly increase the levels of ginsenosides in *Bletilla striata* and flavonoids in *Astragalus mongolicus*, thus indirectly promoting the production of their active pharmacological components (Shi et al., 2023; Li et al., 2024).

Almost 90% of plant species, including flowering plants, bryophytes and ferns, can establish interdependent relationships with AMF (Zhu et al., 2010). AMF form vesicles and arbuscules in the roots, as well as spores and hyphae in the rhizosphere. The extensive hyphal network formed by arbuscular mycorrhizal fungi (AMF) in symbiosis with plant roots significantly increases the volume of soil that the root system can access. This improves the plant’s access to water and essential nutrients, such as phosphorus and nitrogen, thereby promoting growth and development (Bowles et al., 2016). AMF improves plant nutrition by increasing the availability and translocation of various nutrients (Rouphael et al., 2015). AMF improves soil quality by influencing its structure and texture, thereby improving plant health (Zou et al., 2016; Thirkell et al., 2017). Fungal hyphae can expedite the decomposition process of soil organic matter (Paterson et al., 2016). AMF are considered natural growth regulators for most terrestrial flora. They are used as bio-inoculants and researchers encourage their use as bio-fertilizers to promote sustainable crop productivity (Barrow, 2012). Therefore, it is widely believed that AMF could replace inorganic fertilizers in the near future because mycorrhizal application effectively reduces the amount of chemical fertilizer used, especially phosphorus (Ortas, 2012). Moreover, AMF effectively promotes the growth of host plants by increasing the uptake of soil nutrients, especially N and P (Smith et al., 2011). Additionally, Yuan et al. (2023) found that inoculation with arbuscular mycorrhizal fungi (AMF) significantly increased the concentration of active medicinal ingredients. Notably, there was a significant increase in flavonoids.

The cultivation of *Isatis indigotica* has been documented since the Tang dynasty, and it has been grown in various regions of China (Chen et al., 2014; Wong et al., 2022). It is primarily produced in the Chinese regions of Heilongjiang, Gansu, Henan and Hebei, resulting in distinct cultivation germplasm and breeding varieties (Kang et al., 2017; Han et al., 2022). It belongs to the family Cruciferae and is a prevalent Chinese medicinal herb (Zhao, 2007). The roots of *Isatis indigotica* are particularly prized for their high concentration of bioactive compounds, including indole alkaloids, flavonoids and organic acids. These compounds contribute to the plant’s pharmacological efficacy in treating respiratory infections and inflammatory diseases (Chen et al., 2021). Moreover, the roots and leaves have medicinal properties, including the ability to clear heat, detoxify, cool the blood, remove spots, promote the pharynx and relieve pain. They are widely used in the pharmaceutical and food industries (Yu et al., 2021; Su et al., 2023).

The beneficial effects of plant growth-promoting rhizobacteria (PGPR) and arbuscular mycorrhizal fungi (AMF) on crop productivity are widely recognized (Backer et al., 2018; Begum et al., 2019). However, the specific molecular and physiological mechanisms underlying their synergistic interactions with *Isatis indigotica*, as well as the way in which plants respond to PGPR and AMF, are not well understood. The interrelationships between plant agronomic traits, microbiota and metabolites are also largely unknown. In recent years, omics techniques have advanced significantly. Integrating multiple omics datasets has paved the way for a deeper understanding of the interactions between plants and microbes. Multi-omics joint analysis is a new technique that has emerged. The application of plant growth-promoting rhizobacteria (PGPR) and arbuscular mycorrhizal fungi (AMF) in *Isatis indigotica* production is almost non-existent. This study used screening procedures to identify strains related to the growth of *Isatis indigotica*. A multi-omics analysis method was used to comprehensively evaluate the growth indicators, metabolic mechanisms, and rhizosphere microbial of *Isatis indigotica* plants treated with six strains and AMF. This lays the groundwork for the future development and application of these strains.

## 2 Materials and methods

### 2.1 Screening and identification of PGPR

#### 2.1.1 Screening of PGPR

The strains blg1, blg4, blg7, blg9, blg11 and blg16 were isolated from the rhizosphere soil of *Isatis indigotica*. First, 10g of rhizosphere soil is placed in a sterile Erlenmeyer flask and 90 mL of sterile water is added. The flask is then placed in a constant temperature shaker and oscillates at 30°C and 150 rpm for 30min. The resulting solution is diluted 10-fold, and 100 µL of this dilution is spread evenly onto solid Luria Bertani (LB) plates. The inoculated LB plates are incubated at 28°C in a constant temperature incubator for 24h, after which the growth of colonies is observed. After incubation and growth of the colonies, single colonies were selected and purified by multiple streak plating based on various visual characteristics such as color, texture, transparency, size and consistency, as well as other distinct morphological traits. The purified colonies were inoculated onto Luria Bertani (LB) plates and incubated at 28°C overnight. Then, 750 μL of bacterial solution and 750 μL of 50% glycerol were mixed in 2 mL tubes, and stored at −20°C and −80°C as glycerol stocks (Devkota et al., 2024; Jiang et al., 2024).

#### 2.1.2 Identification and validation of PGPR

To identify the bacteria at the molecular level, the 16S rDNA was amplified using PCR with the standard method of the bacterial gene DNA advance kit (Tiangen) (Singh et al., 2015). Genomic DNA was extracted from the strain for subsequent PCR amplification of 16S rDNA using the primers 27F (5’-AGAGTTTGATCCTGGCTCAG-3’) and 1492R (5’- TACGGYTACCTTGTTACGACTT-3’). Perform 50 µL reactions containing 2 µL of each primer, 25 µL of 2× SanTaq PCR Mix (Vazyme), 2 µL of DNA and 19 µL of nuclease-free water, using the PCR system (Bio-Rad). The program used in these PCR assays was as follows: 94°C for 5min, followed by 35 cycles of 95°C for 30 s, 55°C for 30 s, 72°C for 60 s, and 72°C for 10min. The amplification products were sequenced directly and subjected to BLASTN analysis. The sequence alignment of isolated strains was performed using MEGA 7.0 software. Clustering was performed using the neighbor-joining algorithm, and the Kimura two-parameter model was used to calculate evolutionary distances. Node support was determined through bootstrapping, with an estimation conducted using 1,000 replicates (Wang et al., 2024).

### 2.2 Characterization of PGPR isolates

The unique isolates, which were based on morphological features, were tested for six plant growth-promoting (PGP) traits. These included the ability to produce siderophores and indole acetic acid (IAA), to solubilize phosphate (i.e. to facilitate the conversion of organic and inorganic phosphorus), to fix atmospheric nitrogen and to utilize ACC as a nitrogen source (Devkota et al., 2024). For screening of phosphate solubilization, the isolates were patched onto organophosphorus and inorganic phosphorus agar media, respectively, and incubated at 28 °C for 3–5 d (Lin, 2008). The presence of a clear halo around the patch indicated that the isolates were capable of solubilizing phosphate (**Supplementary Figures 3a, b**) (Pikovskaya, 1948). To test their nitrogen-fixing ability, the bacterial isolates were patched onto Norris Glucose Nitrogen-Free Medium and incubated at 28°C for 3–5 d. The presence of a clear halo around the colony indicated nitrogen fixation (**Supplementary Figure 3c**) (Wafula et al., 2020). To test their ability to produce ACC deaminase, the strains were placed on a DF solid medium and incubated at 28°C for 3–5 days, in accordance with the method of Penrose and Glick (2003), with slight modifications. The resulting cultures were then transferred to an ADF medium. Strains producing ACC deaminase were able to grow normally on the medium with ACC as the sole nitrogen source, provided that the same cultivation conditions were maintained (**Supplementary Figure 3d**). An aliquot of 5.0 µL of the bacterial suspension was inoculated onto a CAS agar medium and incubated at 28 °C for 3–5 days. A strain was considered capable of producing siderophores if a yellowish-orange zone appeared around its colonies. Siderophore production was determined using the method described by Wang et al. (1994), with the A/Ar ratio serving as a quantitative indicator of yield. A lower A/Ar ratio indicates higher production, whereas a higher ratio suggests lower production. The bacterial isolates were tested for their ability to produce indole-3-acetic acid (IAA); the isolates were cultured in 5 mL LB broth supplemented with 0.1% tryptophane. The cultures were then incubated at 28°C with continuous shaking at 180 rpm for 48h. An equal volume of non-inoculated LB + tryptophane was used for control. The bacterial growth was sedimented by centrifugation at 10,000 rpm for 10min. Then, 1 mL of the supernatant from each isolate was mixed with 2 mL of Salkowski reagent. The mixture was incubated at room temperature by wrapping with Aluminum foil for 25min. The pink color change indicated IAA production, with the intensity of the color increasing as IAA production increased (**Supplementary Figure 3e**). A mixture of 200 μL of each isolate supernatant and Salkowski reagent was read at 530 nm in triplicate in 96-well round-bottom plates using Spectramax microplate reader (Molecular Devices). To prepare the Salkowski reagent, 2 mL of 0.5 M ferric chloride (FeCl_3_) was dissolved in 49 mL of double-distilled water. Then, 49 mL of 70% perchloric acid (SIGMAALDRICH) was carefully added to the mixture in the chemical hood. An IAA standard curve was prepared by mixing 2 mL of Salkowski reagent with 1 mL of each of the following IAA solution concentrations: 0 g/mL, 5 g/mL, 10 g/mL, 15 g/mL, 20 g/mL, 25 g/mL and 30 g/mL. The equation was then used to calculate the amount of IAA produced by the bacterial isolates (**Supplementary Figure 4**) (Gordon and Weber, 1951).

### 2.3 Experimental design

#### 2.3.1 Preparation of rhizosphere plant growth-promoting bacterial consortium inoculants

The isolated strains were purified using solid LB agar plates. The strains were then cultured in liquid LB medium. To obtain the seed liquid, they were incubated in a shaker at 30°C and 180 rpm for 16 hours. This was then adjusted to an OD_600_ = 1.0. The final artificial synthetic microbial community (ASC) was created by mixing equal quantities of each of the six individual microbial strains in a 1:1:1:1:1:1 ratio. Sterile water was used instead of the microbial mixture for the control group (Jiang et al., 2024).

#### 2.3.2 Pot experiment

The experiment was conducted in a controlled greenhouse environment. *Isatis indigotica* seeds were selected and sterilized with 1% NaClO for five minutes. Then, the seeds were washed three times with sterile water. To accelerate germination, the sterilized seeds were placed in a constant-temperature incubator at 30 °C. Once the germination rate reached 80%, the seedlings were immersed in a microbial consortium consisting of plant growth-promoting rhizobacteria (PGPR) and arbuscular mycorrhizal fungi (AMF), at a ratio of 1:1:1:1:1:1 (w/v), for 16 hours. The seeds were then sown in pots that were 24cm high and had an inner diameter of 21cm. Each pot was filled with 5kg of soil that had been sterilized twice by autoclaving at 121 °C for 40 minutes. The soil mixture was made up of two-thirds local soil and one-third humus. 12g of fresh AMF were inoculated into each pot. In order to maintain a similar microbial community structure in the non-inoculated control groups, the non-inoculated treatment was given 12g of sterilized inoculum and 10 mL of filtered inoculum with a pore size of 0.25 μm.

Thinning was performed when the seedlings reached 5cm in height. A bacterial suspension was applied to the pots every seven days, with the amount adjusted according to the amount of water lost. An equivalent volume of sterile water was used to supplement the control group. All treatments were watered daily with deionized water to maintain soil moisture at 80% of field capacity, except when the bacterial suspension was applied. The aim is to evaluate the effectiveness of combining PGPR with AMF and to provide a more direct reference for its application in the cultivation of medicinal plants. Therefore, the experimental design focuses primarily on evaluating the combined effect of applying PGPR and AMF together, based on the scientific hypothesis that PGPR and AMF have a functional synergistic effect and that applying them together can improve overall agronomic performance by interacting with microbial communities more effectively than a single inoculation. The experimental design included three treatments: (1) the control group (CK), which was treated with water; (2) the PGPR group, which was treated with strains blg1, 4, 7, 9, 11 and 16; and (3) the AMF + PGPR group, which was treated with *Funneliformis mosseae* and strains blg1, 4, 7, 9, 11 and 16. There were 10 replicates for each treatment, with four plants per pot. *Funneliformis mosseae* inoculant used is a standard, viable strain, and the inoculation method (e.g. application to seeds or roots) ensures successful colonization of a wide range of plants (Zhu et al., 2010). After 42 days, the following parameters were measured: plant growth indicators; rhizosphere soil physicochemical properties; microbial community composition; root metabolomic profiles (Cao et al., 2024).

### 2.4 Collection and treatment of samples

The soil samples used in this study were collected from the rhizosphere of *Isatis indigotica* in the greenhouse at Mianyang Normal University in Mianyang City, Sichuan Province, China (31^°^45′ N, 104^°^59′ E). Sampling was conducted in May 2025, with soil samples obtained from the top soil (0–10 cm) in the rhizosphere of the control (CK), PGPR and AMF + PGPR groups. All soil samples were then transferred to sterile containers and stored immediately on dry ice for preservation (Liu et al., 2023; Song et al., 2024). The samples were sieved through 2mm mesh in the lab. Subsequently, the samples were divided into two. One part was air-dried at room temperature to determine some basic physical and chemical indices of the soil. The other 10g of soil samples were stored in sterile tubes and placed in a -80 °C freezer before being sent to Applied Protein Technology Co., Ltd. for bacterial and fungal DNA extraction and high-throughput sequencing. Sterile gloves were worn throughout the sampling process. To eliminate test errors, the Ziploc bag and other sampling tools were sterilized at high temperature. The measurement of each index was completed within 1month after the completion of the sampling (Zhang et al., 2024). In addition, the roots were washed again with sterile water, transferred to 5 mL sterile plastic tubes, and then immediately placed in liquid nitrogen. They were then transported back to the laboratory and stored in a -80°C freezer before being sent to Applied Protein Technology Co., Ltd. for metabolomics sequencing (Cao et al., 2024). The samples were collected in triplicate, i.e., samples were collected from three plants in each treatment as three replicates for each sample (Devkota et al., 2024).

### 2.5 Analysis of the physicochemical properties of rhizosphere soil

The rhizosphere soil of *Isatis indigotica* plants was collected to measure its physical and chemical properties after 42 days. In brief, the pH of the soil solution was measured at soil-to-water ratio of 1:5 using pH meter (Cui et al., 2023). Moreover, the total soil nutrient content was determined using the Kjeldahl method for total nitrogen (TN). Alkaline diffusion was used to measure the soil’s available nitrogen (AN), while the molybdenum-antimony resistance colorimetric method was used to measure the total phosphorus (TP) and available phosphorus (AP) (Tan et al., 2020). Additionally, the soil was pre-treated with K_2_Cr_2_O_7_ and mixed with H_2_SO_4_ to oxidize the organic carbon. The combustion method was then employed to determine the level of soil organic carbon (SOC) (Nishanth and Biswas, 2008; Tan et al., 2020). Three independent experiments were conducted for each sample.

### 2.6 Analyses of bacterial and fungal communities

The rhizosphere soil of *Isatis indigotica* plants was collected after 42 days. Total genomic DNA was extracted from 0.5g of rhizosphere soil using Mag-bind soil DNA kit (Omega) according to the manufacturer’s instructions. The purity and content of the extracted DNA were measured using a spectrophotometer (Nanodrop ND-1000, Thermo Fisher) and checked by agarose gel electrophoresis (1% w/v). The bacterial 16S rDNA (16S V4) was amplified using the forward primer 515F (5’-GTGCCAGCMGCCGCGGTAA-3’) and the reverse primer 806R (5’-GGACTACHVGGGTTWTCTAAT-3’). The fungal ITS was amplified using the forward primer ITS1F (5’-CTTGGTCATTTAGAGGAAGTA-3’) and the reverse primer ITS2-2043R (5’-GCTGCGTTCTTCGATGC-3’) (Wang et al., 2022). All PCR reactions were carried out with Phusion^®^ High-Fidelity PCR Master Mix (New England Biolabs, Inc., Beijing, China), and PCR products were detected by1% agarose gel electrophoresis. Samples containing a bright main strip between 400 and 450 bp were selected and purified using Qiagen gel extraction kit (Qiagen, Dusseldorf, Germany).

Sequencing libraries were generated using the TruSeq^®^ DNA PCR Sample Preparation Kit (Illumina, California, USA), following the manufacturer’s recommendations, and index codes were added. The quality of the libraries was assessed using the Qubit@ 2.0 Fluorometer (Thermo Fisher Scientific Inc., Carlsbad, CA, USA), and the Agilent Bioanalyzer 2100 system (Agilent Technologies, Santa Clara, CA, USA). The libraries were sequenced on an Illumina HiSeq 2500 platform (Shanghai Applied Protein Technology Co. Ltd., Shanghai, China), generating 250 bp paired-end reads. The high-throughput sequencing data were analyzed using Quantitative Insights into Microbial Ecology (QIIME, version 1.8.0) (Caporaso et al., 2010). The same operational taxonomic unit (OTU) was assigned to sequences that were ≥97% similar. The RDP classifier was used to annotate the taxonomic information after the representative sequence for each OTU had been obtained. The ACE, Chao1, Shannon and Simpson indices were used to describe alpha diversity within the bacterial and fungal communities. Beta diversity, which is the difference in bacterial and fungal communities between the three groups, was assessed using principal component analysis (PCoA) (Jiang et al., 2022). Linear discriminant analysis effect size (LEfSe, version 1.0) was used to identify differentially abundant taxonomic features in the rhizosphere bacterial and fungal communities of *Isatis indigotica* in the PGPR, AMF+PGPR, and control (CK) groups (Segata et al., 2011). The *p*-value for the factorial Kruskal–Wallis test was set at 0.05 to identify statistically significant taxonomic biomarkers. A biomarker with a logarithmic LDA score greater than 2.0 was defined as discriminative and visualized.

### 2.7 Extraction of metabolites

The plant roots (80 mg) were frozen immediately in liquid nitrogen and then ground into fine powder using a mortar and pestle. 1000 μL methanol/acetonitrile/H_2_O (2:2:1, v/v/v) were added to homogenized solution for metabolite extraction. The mixture was centrifuged for 20min (14, 000g, 4°C). The supernatant was dried in a vacuum centrifuge. For LC-MS analysis, the samples were redissolved in 100 μL acetonitrile/water (1:1, v/v) solvent and centrifuged at 14, 000g at 4°C for 15min, then the supernatant was injected.

### 2.8 Metabolite analysis using LC-MS/MS

LC-MS/MS analyses were conducted using an UHPLC (1290 Infinity LC, Agilent Technologies) coupled to a quadrupole time-of-flight mass spectrometer (AB Sciex TripleTOF 6600), provided by Shanghai Applied Protein Technology Co., Ltd., Shanghai, China. The samples were separated by Agilent 1290 infinity LC ultra performance liquid chromatography (UHPLC) on a C-18 column; the column temperature was set to 40 °C. The flow rate was set to 0.4ml/min and the injection volume to 2 μL. Mobile phase A consisted of 25 mM ammonium acetate and 0.5% formic acid in water. Mobile phase B consisted of methanol. The gradient elution procedure was as follows: 0-0.5min, 5% B; then B changed to 100% linearly from 0.5 to 10min; 10-12. 0min, B was maintained at 100%; From 12.0 to 12.1min, B changed linearly from 100% to 5%; 12.1–16 min, B was maintained at 5%. Throughout the entire analysis, the sample was kept in an automatic sampler at 4 °C. To avoid the influence of instrument fluctuations, the random sequence was used to analyze the samples. QC samples are added to the sample queue for monitoring and evaluation purposes, to ensure the stability and reliability of the data.

The ESI source conditions were set as follows: Ion Source Gas1 (Gas1) as 60, Ion Source Gas2 (Gas2) as 60, curtain gas (CUR) as 30, source temperature: 600°C, IonSpray Voltage Floating (ISVF) ± 5500V. For MS only acquisitions, the instrument was set to acquire over the m/z range of 60–1,000 Da. The accumulation time for the TOF MS scan was set to 0.20 s per spectrum. For auto MS/MS acquisition, the instrument was set to acquire over the m/z range of 25–1,000 Da and the accumulation time for the product ion scan was set to 0.05 s per spectrum. The product ion scan is acquired using information dependent acquisition (IDA) with high sensitivity mode selected. The parameters were set as follows: the collision energy (CE) was fixed at 35V with ± 15 eV; declustering potential (DP), 60V (+) and −60 V (−); exclude isotopes within 4 Da, candidate ions to monitor per cycle: 10.

### 2.9 Processing of metabolite data

The MS raw data (WIFF scan files) were converted to MzXML files using ProteoWizard MSConvert, and then transferred to the freely available XCMS software for peak alignment, retention time correction and peak area extraction. We set the following parameters to select the peak: centWave m/z = 25 ppm, peak width = c (10, 60) and prefilter = c (10, 100). The following parameters were set to group the peak: bw = 5, mzwid = 0.025 and minfrac = 0.5. Isotopes and adducts were annotated using CAMERA (the Collection of Algorithms of MEtabolite pRofile Annotation). Only variables with non-zero measurements greater than 50% in at least one group were retained among the extracted ion features. Metabolite compounds were identified by comparing the accuracy of the m/z values (within 25 ppm) and the MS/MS spectra with those in an established internal database of reliable, available standards.

### 2.10 Statistical analysis

The soil nutrient content, growth indicators and Alpha diversity statistical analyses in our study were performed using the SPSS 27.0 software (International Business Machines China Co., Ltd., Chengdu, China). One-way ANOVA and the Duncan test were used to analyze the significant differences among groups and determine those based on *p*<0.05. The data were presented as mean ± standard deviation (SD). To gain an understanding of the effects of the PGPR and AMF+PGPR groups, the public web tool (MetaboAnalyst 6.0, https://www.metaboanalyst.ca/MetaboAnalyst/) was used to conduct a PLS-DA (partial least squares discriminant analysis) after log_10_ transformation and autoscaling (Pang et al., 2024). Missing values were replaced by 1/5 of the minimum abundance of respective compounds, assuming that their concentrations were below the detection limit. RDA using Canoco5 software to analyze the relationship between differential accumulation metabolites of roots and dominant rhizosphere microbial genera.

## 3 Results

### 3.1 Plant growth-promoting traits and identification

To obtain the growth-promoting bacteria of *Isatis indigotica*, we isolated them from rhizosphere soil. At least four plant growth-promoting traits were observed in six isolates (**Table 1**). Strains blg4, blg7, blg9, blg11 and blg16 exhibited multiple plant growth-promoting traits, including the ability to solubilize organic and inorganic phosphates, fix nitrogen, produce indole-3-acetic acid (IAA) and exhibit 1-aminocyclopropane-1-carboxylate (ACC) deaminase activity. For example, blg9 exhibits high organic phosphate dissolution efficiency; blg16 demonstrates the greatest capacity for inorganic phosphate dissolution; blg4 exhibits strong nitrogen fixation ability; and blg1 produces high yields of indole-3-acetic acid (IAA). All strains demonstrated considerable competency in these traits. Additionally, blg1, blg7 and blg9 exhibited siderophore biosynthesis. We subsequently conducted morphological analysis and 16S rDNA sequencing of the isolated strains, constructed phylogenetic tree to further identify their taxonomic status. The results showed that all strains exhibited typical bacterial growth characteristics, with some morphological differences observed. The results showed that blg1 was identified as *Acinetobacter* sp., which exhibits white, opaque colonies with smooth surfaces and regular edges. blg7 and blg9 also belonged to *Acinetobacter* sp., with a colony morphology similar to blg1. White, opaque colonies with rough surfaces and irregular edges were displayed by blg4, which was identified as *Bacillus albus*. blg11 and blg16 were identified as *Acinetobacter calcoaceticus*, exhibiting a white, opaque colony with a smooth, moist surface, and regular edges (**Table 1**, **Supplementary Figures 1a-f**, **2a-f**).

**Table 1:** A list of *Isatis indigotica* rhizosphere isolates showing six PGP traits.

Name	OPS	IPS	NF	SP	IAA (μg/mL)	ACC	16S rRNA
blg1	–	2.41 ^± 0.04^	2.20 ^± 0.02^	38.89%	3.54 ^± 0.21^	+	*Acinetobacter* sp.
blg4	2.00 ^± 0.03^	2.11 ^± 0.02^	2.55 ^± 0.03^	–	2.69 ^± 0.03^	+	*Bacillus albus*
blg7	2.24 ^± 0.01^	2.57 ^± 0.06^	1.80 ^± 0.01^	23.73%	3.37 ^± 0.04^	+	*Acinetobacter* sp.
blg9	3.12 ^± 0.05^	3.08 ^± 0.08^	1.89 ^± 0.02^	20.34%	3.03 ^± 0.03^	+	*Acinetobacter* sp.
blg11	1.90 ^± 0.01^	4.13 ^± 0.21^	1.74 ^± 0.05^	–	1.81 ^± 0.01^	+	*Acinetobacter calcoaceticus*
blg16	2.38 ^± 0.05^	6.87 ^± 0.09^	1.48 ^± 0.04^	–	3.03 ^± 0.12^	+	*Acinetobacter calcoaceticus*

‘-’ negative/absent, ‘+’ positive, OPS, Organic phosphorus solubilization; IPS, Inorganic phosphorus solubilization​​; NF, nitrogen fixation; SP, siderophore production; IAA, Indole Acetic Acid production (μg/mL); ACC, ACC deaminase activity. Data represent the SDs of three independent experiments.

### 3.2 Soil properties under the PGPR and AMF+PGPR treatments

The physicochemical properties of rhizosphere soils at the end of the experiment were presented in **Table 2**. The pH and total phosphorus (TP) of rhizosphere soil was fairly stable under CK, PGPR and AMF+PGPR. However, the amount of available nitrogen (AN) in PGPR rhizosphere soils was significantly lower than in CK soils (*p*<0.05). Intriguingly, the amounts of soil organic carbon (SOC), total nitrogen (TN) and available phosphorus (AP) in PGPR and AMF+PGPR rhizosphere soils were significantly higher than that in the control (*p*<0.05).

**Table 2:** Soil physicochemical properties following the application of PGPR and AMF+PGPR inoculant.

Treatment	pH	SOC (g/kg)	TN (g/kg)	TP (g/kg)	AP (mg/kg)	AN (mg/kg)
CK	7.60 ± 0.01b	2.79 ± 1.58c	0.92 ± 0.04c	1.04 ± 0.04a	0.18 ± 0.05b	13.99 ± 3.49a
PGPR	7.89 ± 0.02a	10.54 ± 2.12a	1.11 ± 0.03a	0.97 ± 0.12a	0.75 ± 0.21a	8.16 ± 2.02b
AMF+PGPR	7.52 ± 0.01c	5.51 ± 1.20b	1.00 ± 0.03b	0.94 ± 0.23a	0.52 ± 0.13a	12.83 ± 2.01a

SOC, soil organic carbon; TN, total carbon; TP, total phosphorus; AP, available phosphorus; AN, available nitrogen. CK, water control; PGPR (blg1, 4, 7, 9, 11, 16), AMF+PGPR (*Funneliformis mosseae* + blg1, 4, 7, 9, 11, 16). Data represent the SDs of three independent experiments. Different lowercase letters indicate a significant difference at *p <*0.05. The same lowercase letters indicate non-significant differences.

### 3.3 The effects of PGPR and AMF+PGPR applications on *Isatis indigotica* growth

Since the strains were isolated from the rhizosphere soil of *Isatis indigotica* plants in Guizhou, we first verified their growth-promoting effect. The greatest plant height was achieved with the PGPR treatment alone (*p*<0.05). By contrast, the PGPR+AMF combination produced the most significant improvement in primary and lateral root length, indicating a specific synergistic effect on root system architecture (*p*<0.05). However, the number of leaves was not significantly impacted by PGPR and PGPR+AMF treatment (**Table 3**).

**Table 3:** Growth indicators of *Isatis indigotica* plants following the application of PGPR and AMF+PGPR inoculants.

Treatment	Plant height (cm)	Leaf number	Primary root length (cm)	Lateral root length (cm)
CK	9.27 ± 0.55c	4.67 ± 0.58a	4.46 ± 1.79b	3.66 ± 0.61b
PGPR	40.97 ± 2.33a	6.33 ± 1.52a	4.08 ± 0.72b	2.80 ± 0.39b
AMF+PGPR	34.05 ± 2.59 b	6.00 ± 2.00 a	13.32 ± 3.74a	5.33 ± 0.57a

CK, water control; PGPR (blg1, 4, 7, 9, 11, 16), AMF+PGPR (*Funneliformis mosseae* + blg1, 4, 7, 9, 11, 16). Data represent the SDs of three independent experiments. Different lowercase letters indicate a significant difference at *p <*0.05. The same lowercase letters indicate non-significant differences.

### 3.4 The effects of PGPR and AMF+PGPR applications on rhizosphere microbial community diversity and composition

**Figure 1:**
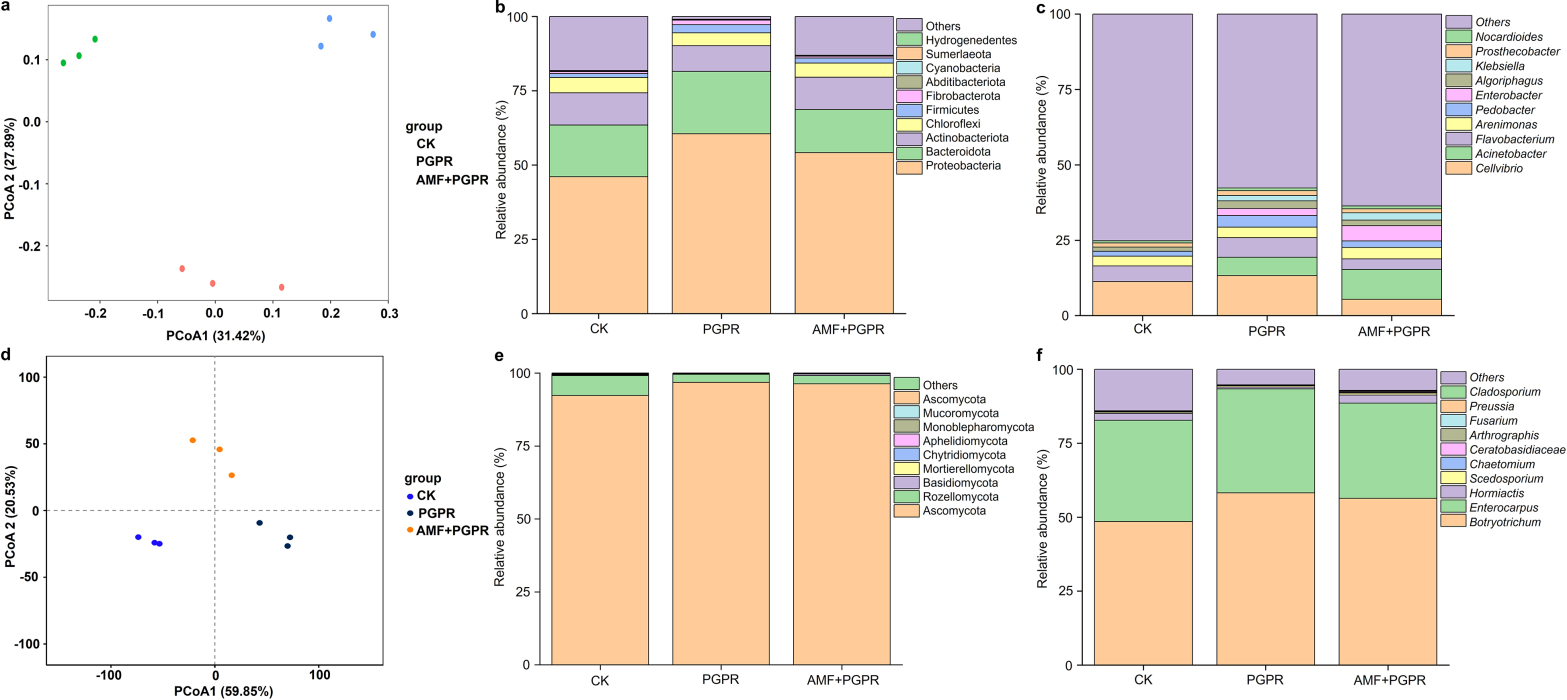
The impact of PGPR and AMF+PGPR on the abundance and composition of bacteria and fungi in rhizosphere soil. **(a, d)** PCoA diagram. Orange and blue circle, control (CK); Green and dark blue circle, PGPR; Light blue and orange circle, AMF+PGPR. X- and Y-axis indicate the first and second principal components (PCoA1 and PCoA2), respectively. Score plots of PCoA1 and PCoA2 show cohesion within each group and separation between the groups, respectively. The relative abundance of bacterial and fungal communities in the rhizosphere soil of *Isatis indigotica* under PGPR and AMF+PGPR treatments at the phylum **(b, e)** and genus **(c, f)** taxonomic levels, respectively. CK, water control; PGPR (blg1, 4, 7, 9, 11, 16), AMF+PGPR (*Funneliformis mosseae* + blg1, 4, 7, 9, 11, 16).

To reveal the diversity and composition of the microbial community in the *Isatis indigotica* rhizosphere, profiles of bacterial and fungal sequencing based on the 16S rRNA and ITS rRNA were generated, respectively. The PCoA scatter diagrams showed that the first (PCoA1) and second (PCoA2) components explained 59.3% and 80.4% of the total variation in the bacterial and fungal communities, respectively. The communities in the rhizosphere soil of the control group (CK) and the treatment groups (PGPR, AMF+PGPR) were independent of each other, indicating that they can be clearly distinguished between the different treatments (**Figures 1a, d**). The alpha diversity indices of the soil microbial community are shown in **Supplementary 1**: **Supplementary Figures S5a**, **S6a**. For bacteria, the difference was significant compared to the control (CK). Both richness (Ace and Chao1) and diversity (Shannon) indices in the treatments were reduced, especially in the PGPR rhizosphere soil (*p <*0.05). For fungi, when compared to CK, AMF+PGPR maintained unchanged richness (Ace and Chao1), while PGPR treatments had significantly decreased values for either index (*p <*0.05). Compared with CK, both Simpson and Shannon diversity indices were significantly decreased under all two treatments (*p <*0.05).

The community barplot analysis also revealed significant alterations in the composition and proportion of bacterial and fungal phyla and genera in rhizosphere soils (**Figures 1b, c, e, f**). The four most predominant bacterial phyla across all samples were the Proteobacteria, Bacteroidota, Actinobacteriota and Chloroflexi (**Figure 1b**), accounting for 85.3% of the relative abundances of all classified bacterial sequences (**Figure 1b**). Proteobacteria was dominant in CK, PGPR and AMF+PGPR soils. In PGPR-treated soil, the highest phylum was Proteobacteria (60.6%), followed by Bacteroidota (21.0%). Furthermore, the relative abundance of Actinobacteriota and Chloroflexi increased in the CK- and AMF+PGPR-treated samples (**Figure 1b**). At genus level, the five most predominant bacteria present in soil across all samples were *Cellvibrio*, *Acinetobacter*, *Flavobacterium*, *Arenimonas* and *Pedobacter* (**Figure 1c**). Among these five genera, the rhizosphere soil under the PGPR and AMF+PGPR treatments had the highest level of *Acinetobacter* compared to CK (**Figure 1c**).

Of the classified fungal community (**Figure 1e**), Ascomycota and Rozellomycota phyla were the two most abundant, together accounting for 99.2% of total fungal sequence relative abundance. The most abundant phylum Ascomycota was enriched the most in PGPR and AMF+PGPR (96.5%), and diminished the most in CK (92.2%) treatment. Interestingly, the second-ranked phylum, Rozellomycota, was found to be more abundant under the CK treatment. At the genus level (**Figure 1f**), the five most predominant fungi across all samples were *Botryotrichum*, followed by *Enterocarpus*, *Hormiactis*, *Scedosporium* and *Chaetomium*. Among them, *Botryotrichum*, *Enterocarpus* and *Scedosporium* were found to reach higher levels across the PGPR and AMF+PGPR treatments than the CK. Notably, *Hormiactis* and *Chaetomium* were increased in AMF+PGPR-treated soils in comparison to the CK.

The cladogram, which illustrates evolutionary relationships and biodiversity between species, revealed significant differences in rhizosphere microbiota between the PGPR, AMF+PGPR and CK groups (**Supplementary Figures 5b**, **6b**). Lefse analysis of biomarkers showed that *Devosia*, *Algoriphagus*, *Methylotenera*, *Dyadobacter*, *Stenotrophomonas*, *Pseudohongiella*, *Paenibacillus*, *Marmoricola*, *Caulobacter*, *Persicitalea*, *Glutamicibacter*, *Cohnella*, *Sphingorhabdus*, *Candidimonas*, *Achromobacter*, *Glycomyces*, *Larkinella*, and *Enterobacter*, *Klebsiella*, *Latescibacterota*, *Nitriliruptoraceae*, *Latescibacteraceae*, *Erythrobacter*, *Antarcticibacterium*, and *Melanospora*, *Berkeleyomyces*, *Ceratobasidiaceae_gen_Incertae_sedis*, *Epicoccum*, *Tausonia*, *Talaromyces*, *Chaetomium*, *Acaulium* were the biomarkers in the rhizosphere soil of the PGPR and AMF+PGPR strains, but not in the CK (**Supplementary Figures 5c**, **6c**), suggesting that these bacteria and fungi were recruited to the *Isatis indigotica* rhizosphere by the PGPR and AMF+PGPR strains.

### 3.5 Metabolic differences of *Isatis indigotica* roots under the PGPR and AMF+PGPR treatments

**Figure 2:**
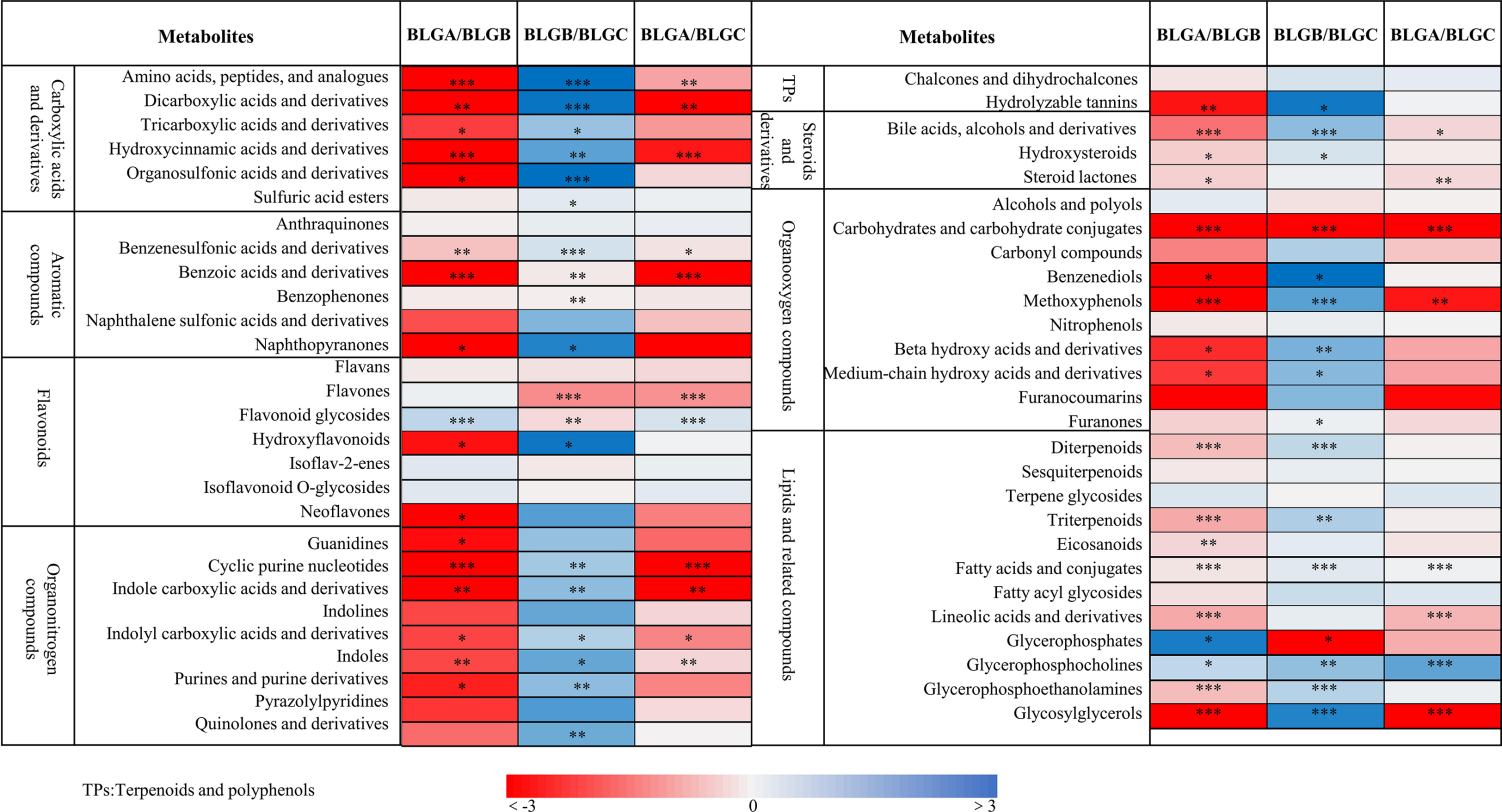
Fold change (log2) of carboxylic acids and their derivatives, aromatic compounds, flavonoids, and organic nitrogen compounds, steroids and derivatives, organicoxygen compounds, lipids and related compounds, and other compounds in the roots of *Isatis indigotica* were analyzed under 42 days of inoculation with PGPR and AMF+PGPR. CK (BLGA), PGPR (BLGB), AMF+PGPR (BLGC). Asterisks indicate significant differences between the control (BLGA, CK) and treatments (BLGB, PGPR; BLGC, AMF+PGPR) within the same metabolite (*p<0.05; **p<0.01; ***p<0.001). Data represent the SDs of three independent experiments.

PGPR and AMF+PGPR inoculation significantly affected the content of 8 categories of metabolites in the roots of *Isatis indigotica*, including carboxylic acids and derivatives, aromatic compounds, flavonoids, organonitrogen compounds, terpenoids and polyphenols, steroids and derivatives, organooxygen compounds, and lipids and related compounds (**Figure 2**). Compared with CK (BLGA), inoculation with PGPR (BLGB) and AMF + PGPR (BLGC) significantly increased the abundances of the following metabolites: amino acids, peptides and analogues; dicarboxylic acids and derivatives; hydroxycinnamic acids and derivatives; organosulfonic acids and derivatives; benzenesulfonic acids and derivatives; benzoic acids and derivatives; cyclic purine nucleotides; indole carboxylic acids and derivatives; indolyl carboxylic acids and derivatives; indoles; bile acids, alcohols and derivatives; steroid lactones; carbohydrates and carbohydrate conjugates; methoxyphenols; fatty acids and conjugates; linolenic acids and derivatives; and glycosylglycerols (*p*<0.05, *p*<0.01, or *p*<0.001). Meanwhile, abundances of following metabolites: tricarboxylic acids and derivatives; organosulfonic acids and derivatives; naphthopyranones; hydroxyflavonoids; neoflavones; guanidines; purines and purine derivatives; hydrolysable tannins; hydroxysteroids; benzenediols; beta hydroxy acids and derivatives; medium-chain hydroxy acids and derivatives; diterpenoids; triterpenoids; eicosanoids and glycerophosphoethanolamines were also significantly increased by inoculation with PGPR (BLGB) compared to CK (BLGA) (*p <*0.05, *p*<0.01 or *p*<0.001). And flavones abundances increased significantly inoculation with AMF + PGPR (BLGC) (*p*<0.001). However, inoculation with both PGPR (BLGB) and AMF + PGPR (BLGC) significantly decreased the abundances of flavonoid glycosides and glycerophosphocholines (*p*<0.05 or *p*<0.001), while inoculation with PGPR (BLGB) also significantly decreased the abundance of glycerophosphates (*p*<0.05) (**Figure 2**).

**Figure 3:**
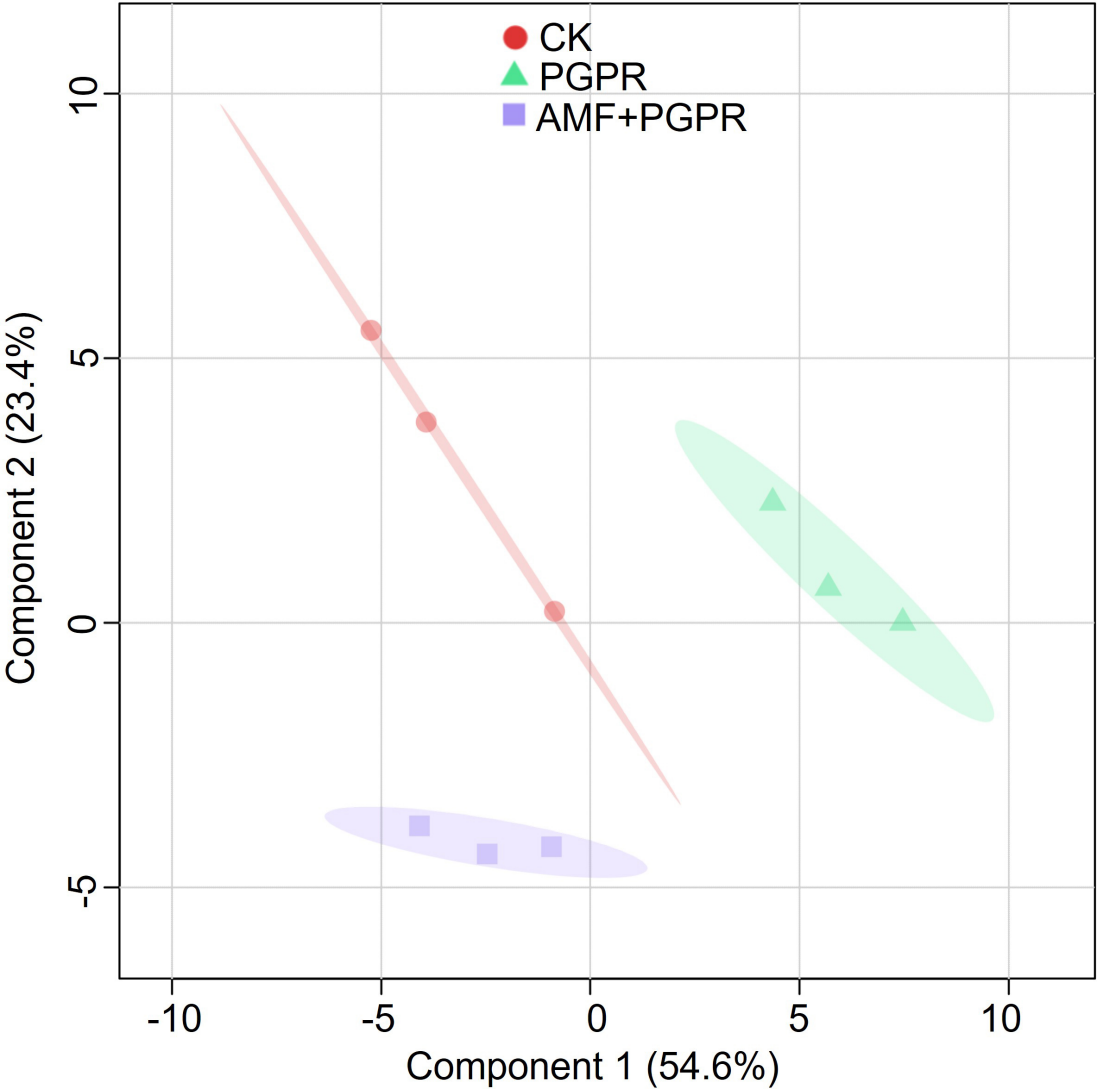
Cluster analysis of all metabolic parameters of *Isatis indigotica* roots. The shapes (circles, triangles, and squares) represent the CK, PGPR, and AMF+PGPR treatments, respectively. Semi-transparent shadings indicate 95% confidence regions.

**Figure 4:**
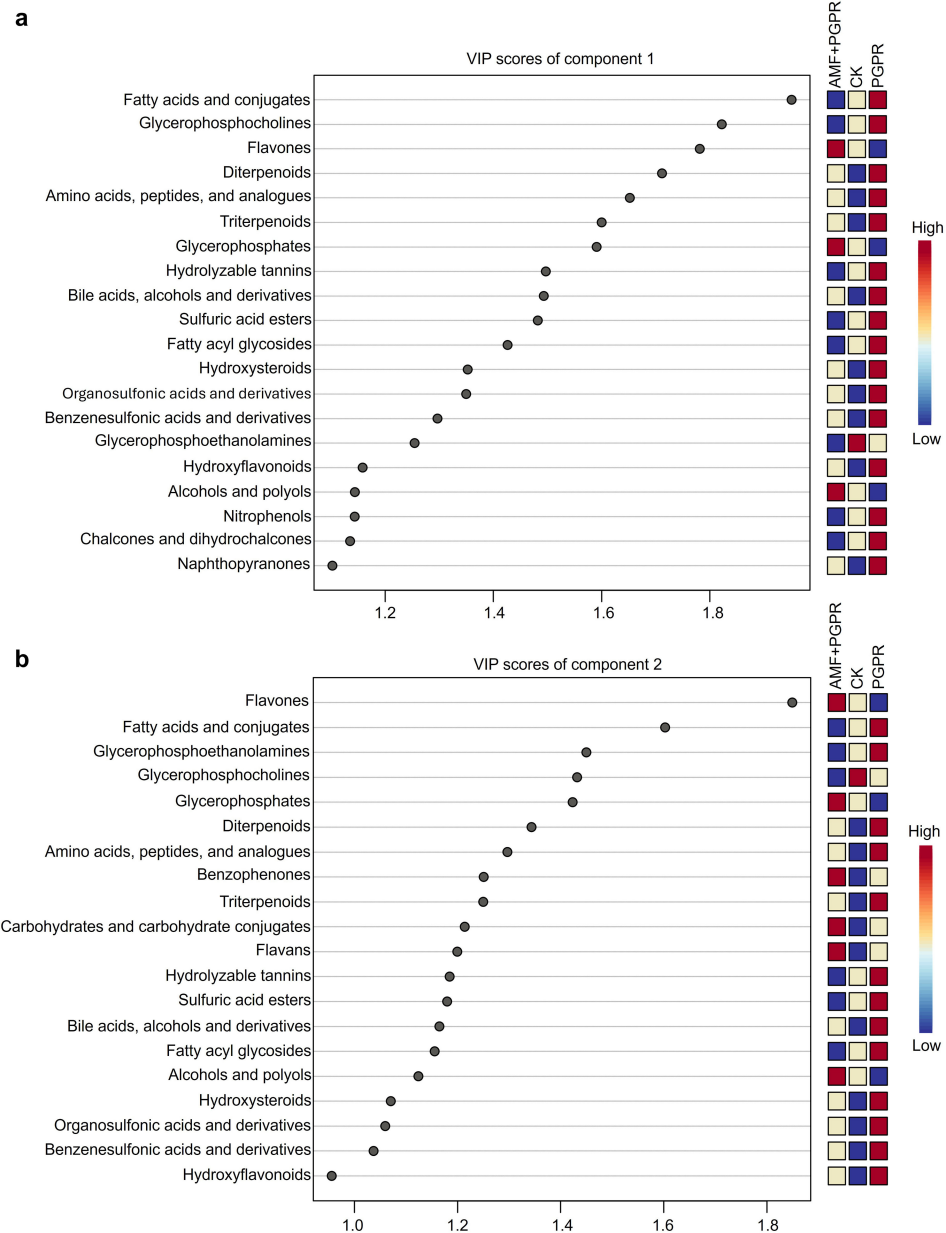
The top 20 parameters of components 1 and 2, according to the VIP scores of the PLS-DA analysis, are shown in the upper **(a)** and lower **(b)** panels.

PLS-DA analysis was performed based on all metabolite parameters for inoculations with different bacterial agents (R² = 0.93, Q² = 0.63; **Figure 3**). The PGPR effect is separated along component 1, while the AMF+PGPR specific effect is mainly separated along component 2. These two components together explain 79.1% of the variation, with the fatty acids, conjugates; flavonoids; glycerophosphocholines and glycerophosphoethanolamines largely determined the two components (**Figure 4**).

### 3.6 Redundancy analysis of accumulated metabolites, and dominant microbial genera, growth indicators under PGPR and AMF+PGPR treatments

**Figure 5:**
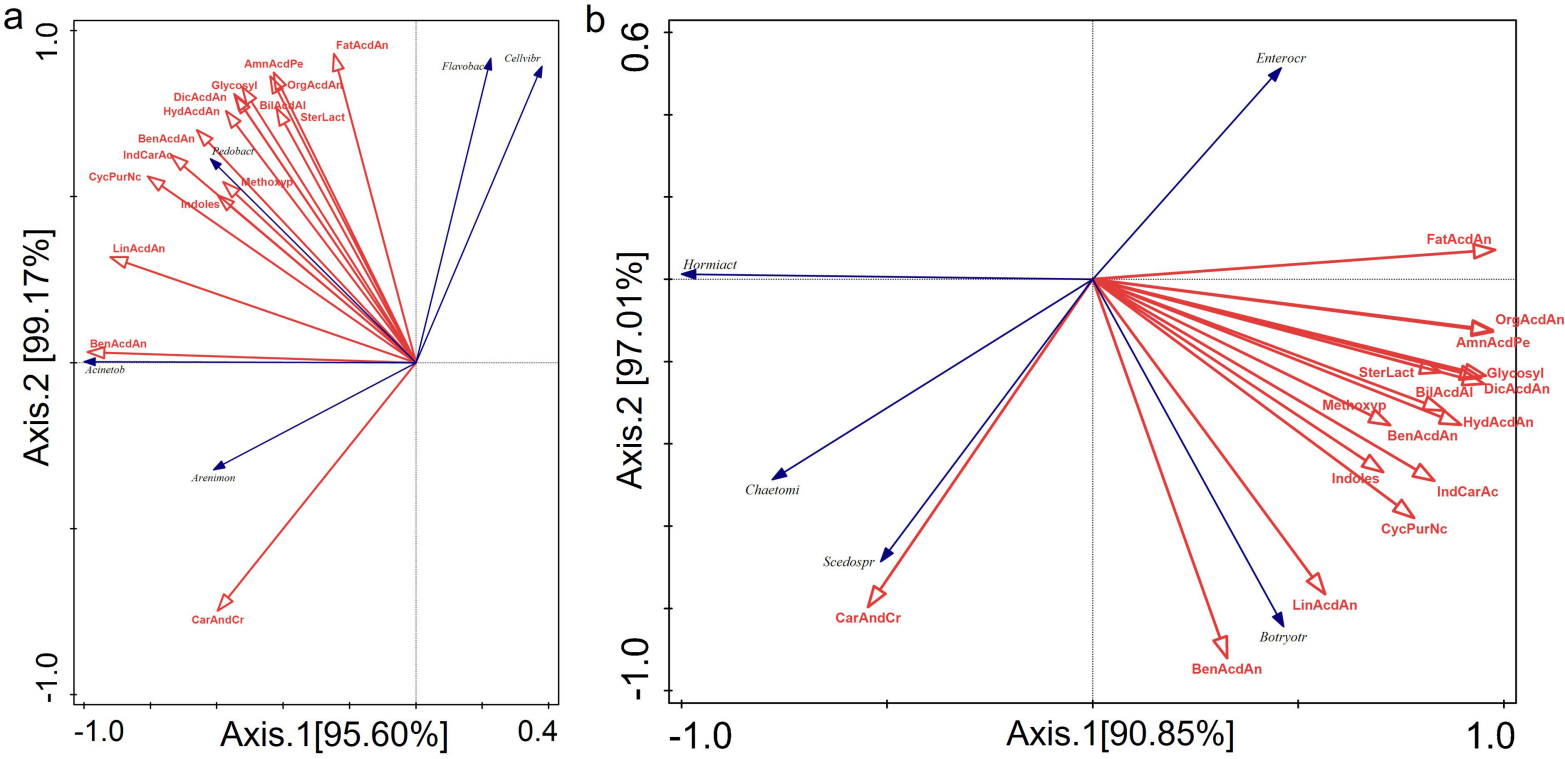
Redundancy analysis (RDA) was performed to investigate the relationship between the dominant bacterial genus **(A)** and the fungal genus **(B)**, as well as the relationship between the differentially accumulated metabolites. The full names of the bacterial genera abbreviated in figure A are Cellvibrio, Acinetobacter, Flavobacterium, Arenimonas and Pedobacter. The full names of the abbreviated fungal genera in figure B are Botryotrichum, Enterocarpus, Hormiactis, Scedosporium and Chaetomium. The full names of the abbreviated metabolites in the figure are: Amino acids, peptides and analogues; dicarboxylic acids and derivatives; hydroxycinnamic acids and derivatives; organosulfonic acids and derivatives; benzenesulfonic acids and derivatives; benzoic acids and derivatives; cyclic purine nucleotides; indole carboxylic acids and derivatives; indoles; bile acids, alcohols and derivatives; steroid lactones; carbohydrates and carbohydrate conjugates; methoxyphenols; fatty acids and conjugates; linolic acids and derivatives; glycosylglycerols.

Redundancy analysis (RDA) indicates a strong correlation between differentially accumulated metabolites and rhizosphere microbial communities. The cumulative contribution rate of the first two axes in the correlation analysis between dominant bacterial genera and differentially accumulated metabolites is 99.17%. There is a strong correlation between carbohydrates and carbohydrate conjugates and *Arenimonas*. Benesulfonic acids and derivatives impact *Acinetobacter*, while other metabolites significantly impact *Pedobacter* (**Figure 5a**). Additionally, the cumulative contribution rate of the first two axes in the correlation analysis between dominant fungal genera and differentially accumulated metabolites was 97.01%. Carbohydrates and carbohydrate conjugates significantly impact *Scedosporium*. Fatty acids and conjugates impact *Enterococcus*. Benesulfonic acids and derivatives, as well as linelic acids and derivatives, strongly correlate with *Botryotrichum* (**Figure 5b**).

## 4 Discussion

In this study, six strains of *Isatis indigotica* PGPR and one AMF (*Funneliformis mosseae*), including 3 strains of *Acinetobacter* sp. (blg1, blg7 and blg9), 2 strains of *Acinetobacter calcoaceticus* (blg11 and blg16), and 1 strain of *Bacillus albus* (blg4), were used to make a combined bacterial agent. It was revealed that the application of a combined microbial inoculant of PGPR and AMF had a significant effect on plant growth in a pot experiment. This finding is consistent with the results of numerous previous studies that have demonstrated the beneficial effects of PGPR and AMF+PGPR strains on plant growth and development (Adesemoye et al., 2008; Chatzistathis et al., 2024).

The isolated bacterial strains (blg1, blg4, blg7, blg9, blg11, and blg16) exhibited a variety of plant growth-promoting (PGP) traits, such as phosphate solubilization, nitrogen fixation, indole-3-acetic acid (IAA) production and 1-aminocyclopropane-1-carboxylate (ACC) deaminase activity (**Table 1**). These traits are essential for enhancing nutrient availability and stress resilience in plants (Trivedi et al., 2020; Sokol et al., 2022). Notably, *Acinetobacter* sp. (blg1, blg7, blg9, blg11 and blg16) exhibited robust IAA production and phosphate solubilization (**Table 1**). This aligns with previous reports indicating that *Acinetobacter* strains promote plant growth by modulating phytohormones and mineralizing nutrients (Rokhbakhsh-Zamin et al., 2011; Josephine and Thomas, 2022). The *Acinetobacter calcoaceticus* species help to solubilize recalcitrant phosphate in soil, which promotes phosphate availability to plants (Ishaq et al., 2025). The superior nitrogen-fixing ability of *Bacillus albus* (blg4) (**Table 1**) further supports its role in improving soil nitrogen content, which is essential for plant biomass accumulation (Bhattacharyya and Jha, 2012; Aasfar et al., 2024). It has been reported that *Bacillus albus* (DS9) exhibits characteristics of drought stress tolerance and plant growth promotion (Ashry et al., 2022).

Significant increases in SOC and AP were observed in soils treated with PGPR and AMF (**Table 2**), suggesting that these microbes enhance nutrient cycling and the decomposition of organic matter. Moreover, co-inoculation of PGPR and AMF significantly promoted root growth. This may encourage the roots to become more active and act as the main carbon pump. This process involves the secretion of large quantities of unstable organic compounds, such as sugars and organic acids, which promote root turnover and directly supply fresh, easily metabolized carbon to the soil. The local microbial community, stimulated by PGPR, experienced rapid proliferation. This growth and subsequent turnover significantly contributed to the soil organic carbon (SOC) pool through the accumulation of microbial biomass carbon (MBC) and dead microbial matter. Furthermore, after PGPR inoculation, the amount of available nitrogen (AN) decreased sharply, while the total nitrogen (TN) content increased moderately (**Table 2**). This may be due to the rapid growth of the PGPR-inoculated microbiota, which requires large amounts of nitrogen for the synthesis of cellular components. Therefore, microorganisms absorb and convert readily available inorganic nitrogen into organic nitrogen within their biomass, resulting in a reduction in nitrogen levels. An increase in TN may indicate the contribution of accumulated microbial biomass nitrogen to the soil nitrogen pool. This is consistent with studies showing that microbial inoculants improve soil fertility by stimulating microbial activity (Ephraim, 2023; Han et al., 2025). The lack of variation in pH across the different treatments (**Table 2**) suggests that the microbial inoculation did not affect the acidity of soil, which is important for maintaining the stability of rhizosphere (Pantigoso, 2022). In addition, PGPR alone primarily promotes growth in terms of plant height. In contrast, the combined inoculation synergistically enhances the development of root system architecture, i.e. the elongation of primary and lateral roots (**Table 3**). This suggests that PGPR may stimulate above-ground growth directly by producing phytohormones (e.g. auxins), whereas AMF enhances the absorption capacity of roots by expanding the hyphal network. Furthermore, PGPR and AMF are functionally complementary: PGPR promotes above-ground growth while working with AMF to optimize root architecture. This significantly improves the plant’s ability to absorb soil nutrients (Pérez-de-Luque et al., 2017). These findings were confirmed by the study of Visen et al. (2021), which showed that the concurrent inoculation of *Litchi chinensis* S. with AMF and PGPR resulted in maximum root colonization and plant growth.

Microbial communities and their composition can vary structurally in response to microbial inoculant applications (Fageria and Baligar, 2005). Indeed, we found that the diversity and abundance of the PGPR and AMF+PGPR treatments differed most from the control group (**Figure 1**, **Supplementary Figures 5a**, **6a**). The dominance of Proteobacteria in PGPR- and AMF+PGPR-treated soils (**Figure 1b**) may be due to an elevated concentration and diversity of soil resources, which could regulate competitive species interactions within this phylum and enhance its abundance (Xiao et al., 2024). Recently, Kong et al. (2025) reported that the treatment of *Populus simonii Carr* with AMF+*Bacillus subtilis* (BM) resulted in a significant increase in the relative abundance of Proteobacteria in soil, compared to CK. The results reported by Chen et al. (2025) also indicate that Proteobacteria are the most dominant phylum within the tobacco rhizosphere bacterial community under AMF application. Moreover, the increased relative abundance of Bacteroidota in PGPR-treated soil (**Figure 1b**) could be linked to their role in organic matter degradation (Hou et al., 2015). Zou et al. (2024) recently reported that co-applying BC and PGPR to Chinese cabbage increased the relative abundance of Bacteroidetes in soil compared to the control. The enrichment of *Acinetobacter* in PGPR-treated soils (**Figure 1c**) corroborates findings by Nihorimbere et al. (2011), who highlighted its role in biocontrol and nutrient mobilization. Similarly, the increased abundance of *Botryotrichum*, *Enterocarpus* and *Scedosporium* in PGPR- and AMF-treated soils (**Figure 1f**) may be due to their synergistic interactions with beneficial bacteria. Notably, the rise of *Chaetomium* and *Hormiactis* in AMF+PGPR-treated soils may reflect their biocontrol potential against soil-borne pathogens (Sharma et al., 2025). The LEfSe analysis identified multiple biomarkers (e.g., *Devosia*, *Paenibacillus*, *Talaromyces*) in PGPR- and AMF+PGPR-treated soils (**Supplementary Figures 5c**, **6c**), may reinforce the concept of microbial recruitment by plant-beneficial inoculants. These taxa have been previously associated with nitrogen fixation (*Devosia*), chitin degradation (*Paenibacillus*), and secondary metabolite production (*Talaromyces*) (Rivas et al., 2003; Itoh et al., 2013; Lei et al., 2022). Our findings support the hypothesis that PGPR and AMF synergistically reshape the rhizosphere microbiome, enhancing the abundance of beneficial taxa while suppressing pathogens.

The composition of the plant-associated microbiome is influenced by a variety of factors, such as the host’s genotype, root morphology and root exudates (Sasse et al., 2018). In turn, changes in plant metabolic profiles caused by microbial inoculations might have an impact on the patterns of root exudation (Kong and Liu, 2022). We found that the profound restructuring of root metabolites observed in *Isatis indigotica* following inoculation with PGPR and AMF+PGPR reveals complex metabolic interactions between plants and microbes with significant physiological implications. The increase in indole carboxylic acids and derivatives in both treatments (*p*<0.001) (**Figure 2**) may reflect the regulation of root development and stress response (Kudoyarova et al., 2019). This is consistent with the results of Kudoyarova et al. (2017), who found that inoculating wheat plants with the auxin-producing bacteria *Bacillus subtilis* IB 1087 and *Pseudomonas* IB-K13–1 resulted in an increase in root mass. The significant increase in the abundances of key metabolite classes, including amino acids, flavonoids and polyphenols (**Figure 2**), showed that microbial inoculants activate multiple biosynthetic pathways simultaneously (Saia et al., 2015; Gasemi et al., 2023; Ferreira et al., 2024). Moreover, flavonoids and fatty acids contributed significantly to the clustering of the two treatments, as shown in the VIP scores plot of the top 20 parameters (**Figure 4**). Recent research has shown that treating A. *mongholicus* with a combination of bacterial preparations significantly increases the amounts of flavonoid metabolites in the plant’s root tissues. Additionally, the accumulation of three triterpenoid saponin metabolites, along with some amino acids and other substances, was significantly increased (Shi et al., 2024). Of particular note is the differential regulation of specialized metabolites: PGPR was found to specifically enhance hydroxyflavonoids (*p*<0.01) and neoflavones (*p*<0.001), while AMF + PGPR was found to uniquely upregulate flavones (*p*<0.001) (**Figure 2**). This suggests that these microbial partners regulate plant secondary metabolism in different ways (Pang et al., 2021). Previous research has shown that the flavonoid content of tobacco plants increases with AMF inoculation and PGPR, either alone or in combination (Begum et al., 2022). Furthermore, both treatments resulted in an increase in linolenic acid derivatives (*p*<0.01) and a decrease in glycerophosphocholine (*p*<0.05), as shown in **Figure 2**. This suggests that PGPR and AMF+PGPR activate the membrane adaptation stress tolerance response in the root system of *Isatis indigotica*. The former improves membrane fluidity and transmits stress resistance signals, while the latter reflects the rapid turnover of phospholipids necessary to maintain membrane homeostasis (Savchenko et al., 2014; Keymer et al., 2018).

It has been shown that the compounds released by the root system attract beneficial microorganisms and influence the composition of the rhizosphere microbiome, thereby enhancing the plant’s ability to adapt to its environment (Bulgarelli et al., 2013). Redundancy analysis (RDA) reveals a remarkably strong correlation between the structure of the rhizosphere microbial community and the root metabolite profile of *Isatis indigotica* under PGPR, PGPR and AMF inoculation. The exceptionally high cumulative contribution rates of the first two RDA axes (99.17% for bacteria and 97.01% for fungi) (**Figures 5a, b**) suggest that shifts in the dominant microbial genera are largely explained by changes in specific root metabolites. More importantly, other metabolites significantly impact *Pedobacter* (**Figure 5a**), which suggest that this bacterium may act as a keystone species, integrating multiple metabolic pathways. This is consistent with its established role as a plant growth promoter (Kudoyarova et al., 2019). Additionally, Carbohydrates and carbohydrate conjugates significantly impact *Scedosporium* (**Figure 5b**). This metabolic specialization may explain how they coexist in the rhizosphere by partitioning resources (Jacoby and Kopriva, 2019). These findings suggest that the inoculated beneficial microbes not only coexist with the plant, but also actively contribute to the formation of a distinct rhizosphere microenvironment by interacting with, or inducing the production of, specific root exudates.

One limitation of this study is that PGPR were screened and cultivated in this study were mainly identified at genus level based on 16S rRNA gene sequences. Although this study provides an important foundation for understanding the beneficial microbial resources with potential applications in the rhizosphere of *Isatis indigotica*, future research will use techniques such as multi-site sequence analysis or whole genome sequencing to accurately identify key growth-promoting strains, such as *Acinetobacter* and *Bacillus albus*, and elucidate their functional mechanisms. Another, limitation of this study is that it did not include a treatment inoculated with AMF alone. This omission makes it more difficult to attribute the observed effects precisely, particularly when it comes to discerning the individual contribution of AMF from the synergistic interaction with PGPR. Incorporating separate AMF treatments into future research would provide key insights into precisely analyzing the independent contributions and interaction mechanisms of AMF and PGPR in promoting plant growth. While the use of three biological replicates per treatment is consistent with initial exploratory omics studies, it may limit the statistical power to detect nuanced interactions. Further research involving larger groups would be valuable in validating these findings and revealing more subtle effects. Furthermore, it was not possible to directly quantify AMF root colonization rates. Future studies that incorporate microscopic assessment or molecular quantification of fungal colonization are essential for definitively correlating the observed synergistic growth effects with the extent of symbiosis establishment. Although our RDA analysis revealed strong correlations between specific microbial taxa and root metabolites, future studies using co-occurrence network analysis are essential for understanding the complex interspecies interactions of the rhizosphere microbiome under PGPR and AMF inoculation. Moreover, future work will prioritize the use of qPCR with strain-specific primers in order to monitor the dynamics of colonization and persistence of the key inoculated strains identified in this study with precision.

## 5 Conclusion

Our study revealed functional differences between PGPR (e.g. *Acinetobacter* sp. and *Bacillus albus*) and AMF in their ability to promote the growth of *Isatis indigotica*. A single inoculation of PGPR primarily promotes plant height, whereas co-inoculation with AMF optimizes root structure synergistically (i.e. length of primary and lateral roots). This significantly increases the efficiency with which plants obtain soil nutrients (SOC, TN and AP). Analysis of the microbiome revealed the selective enrichment of beneficial taxa, such as Proteobacteria and Ascomycota. This suggests that plants may actively recruit these beneficial microorganisms. Metabolomic profiling revealed that inoculation altered several key metabolites, including amino acids, flavonoids and lipids. Redundancy analysis (RDA) confirmed strong correlations between key microbial genera (e.g., *Acinetobacter*, *Pedobacter*, and *Botryotrichum*) and the accumulation of distinct root metabolites, such as benzenesulfonic acids, carbohydrates, and fatty acids. These changes are likely to promote plant growth synergistically and enhance stress resilience. Overall, our findings clarify how PGPR and AMF alter the rhizosphere microbiome and root metabolite profiles. This provides a theoretical basis for the development of biofertilizers for the cultivation of medicinal plants.

## Data Availability

The original contributions presented in the study are included in the article/**Supplementary Material**. Further inquiries can be directed to the corresponding author.
