# Supplementary figures and images for: Integrated omics analysis of PGPR and AMF effects on soil microbiota and root metabolites in Isatis indigotica
Source: Front Microbiomes. 2025 Nov 17;4:1709335. doi: 10.3389/frmbi.2025.1709335 (PMC12993667; doi:10.3389/frmbi.2025.1709335)

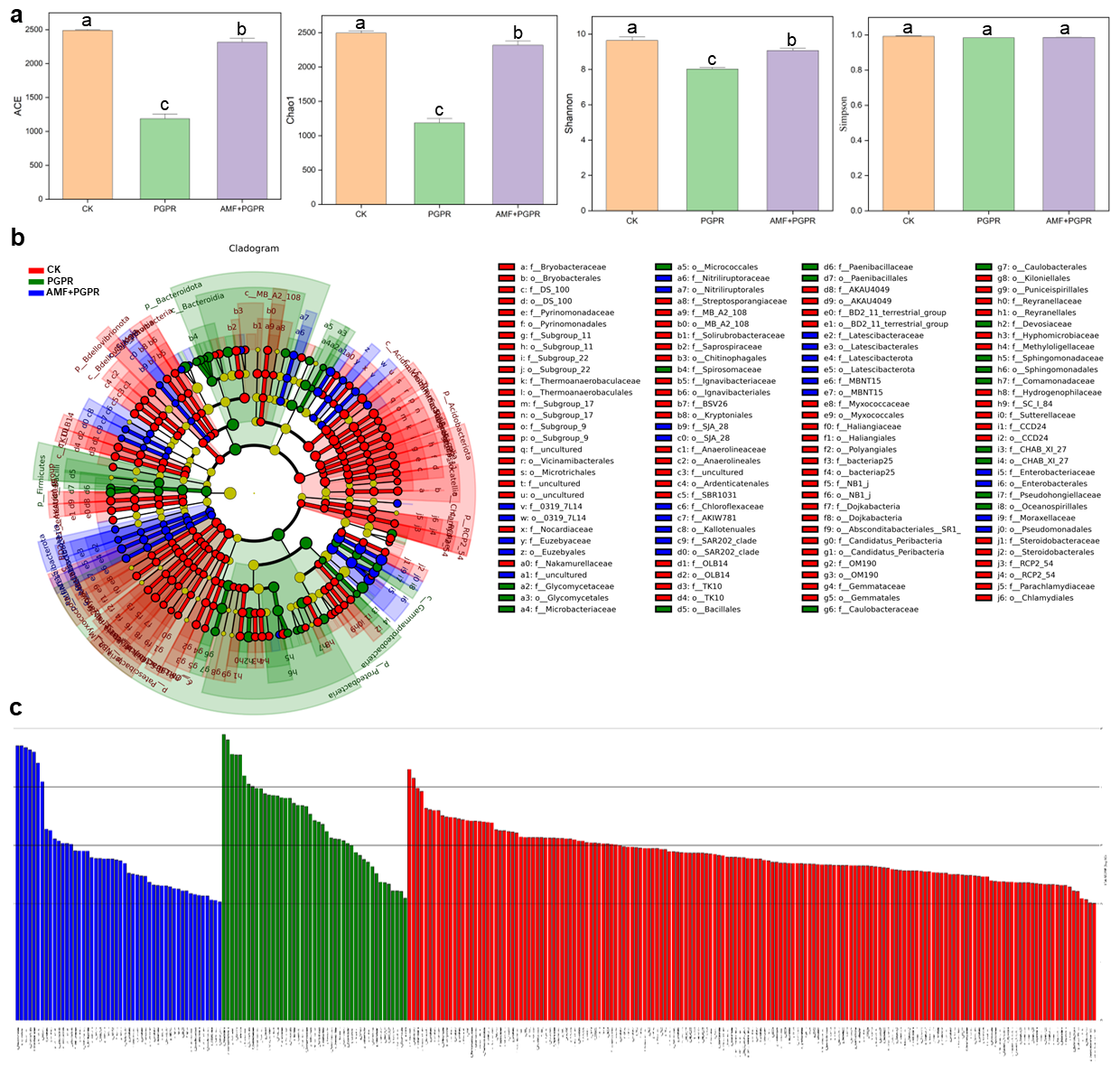

Supplement: Supplementary file 1 [file Image5.tif]

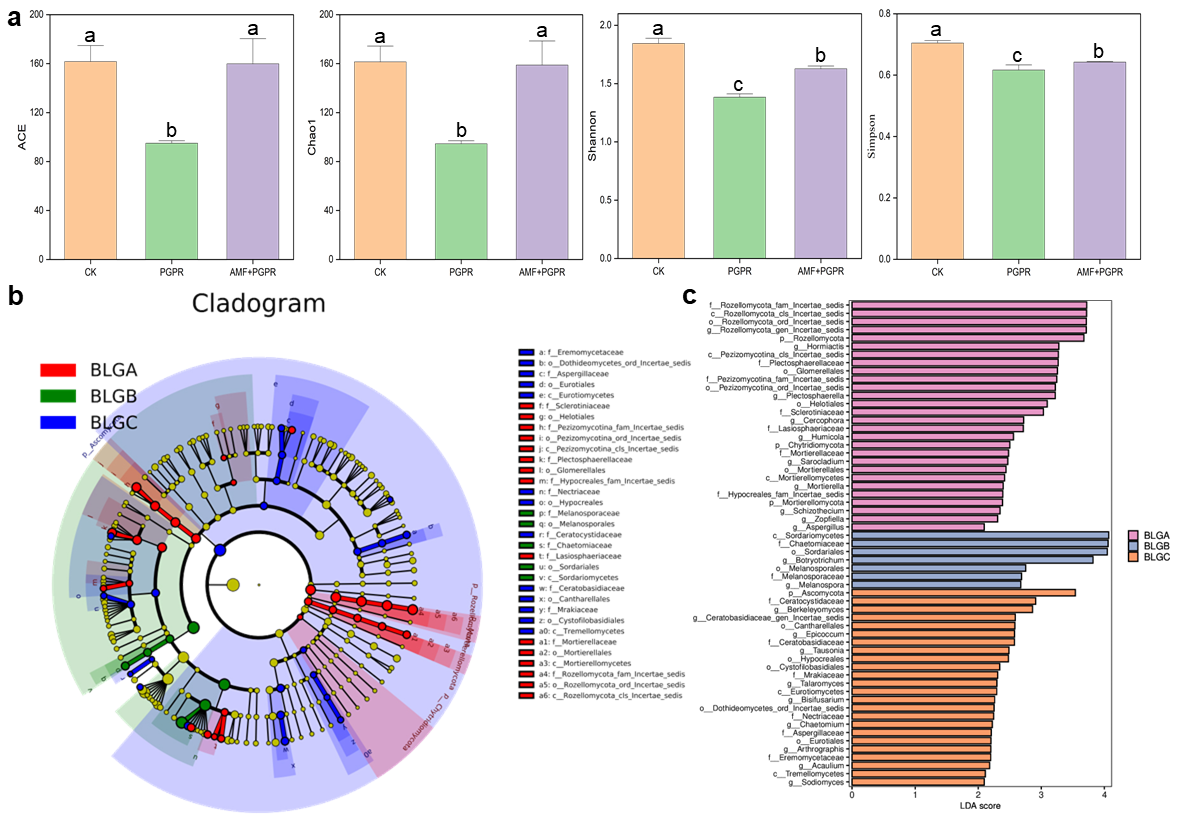

Supplement: Supplementary file 2 [file Image6.tif]
