# Supplementary material for: Integrated omics analysis of PGPR and AMF effects on soil microbiota and root metabolites in Isatis indigotica
Source: Front Microbiomes. 2025 Nov 17;4:1709335. doi: 10.3389/frmbi.2025.1709335 (PMC12993667; doi:10.3389/frmbi.2025.1709335)
Supplement: Supplementary file 3 [file Supplementaryfile1.docx]

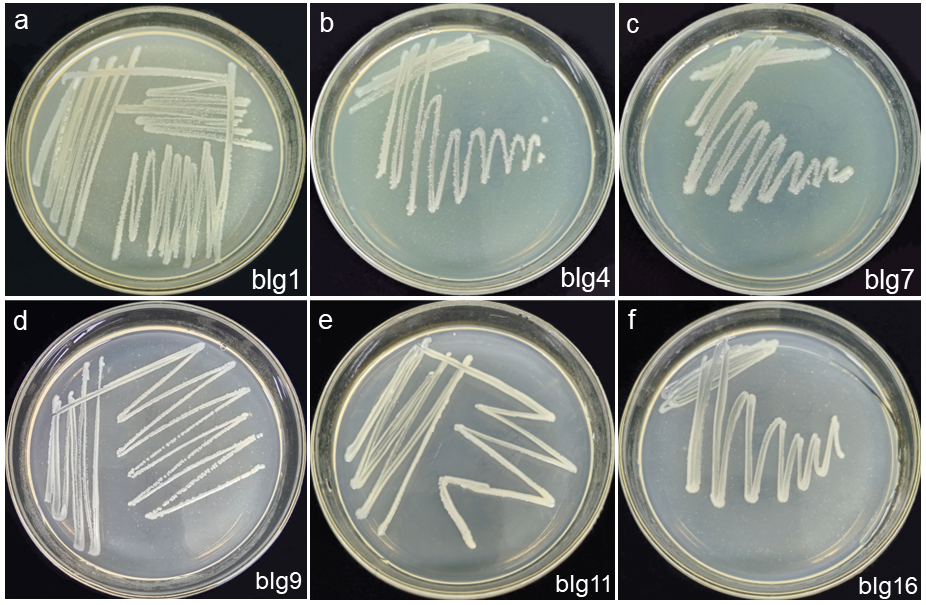


**Supplementary Figure 1** Morphological characteristics of the strain blg1 (**a**), blg4 (**b**), blg7 (**c**), blg9 (**d**), blg11 (**e**) and blg16 (**f**) in the dishes.

**Supplementary Figure 2** Identification and characteristics of the PGPR strain. (**a**-**f**) Phylogenetic tree of strain blg1, blg4, blg7, blg9, blg11 and blg16, respectively. The genus of the strains blg1, blg4, blg7, blg9, blg11 and blg16 was identified using 16S rDNA sequencing. MEGA 7.0 was used to construct the phylogenetic tree. The neighbour-joining method was used with 1000 bootstrap replications.


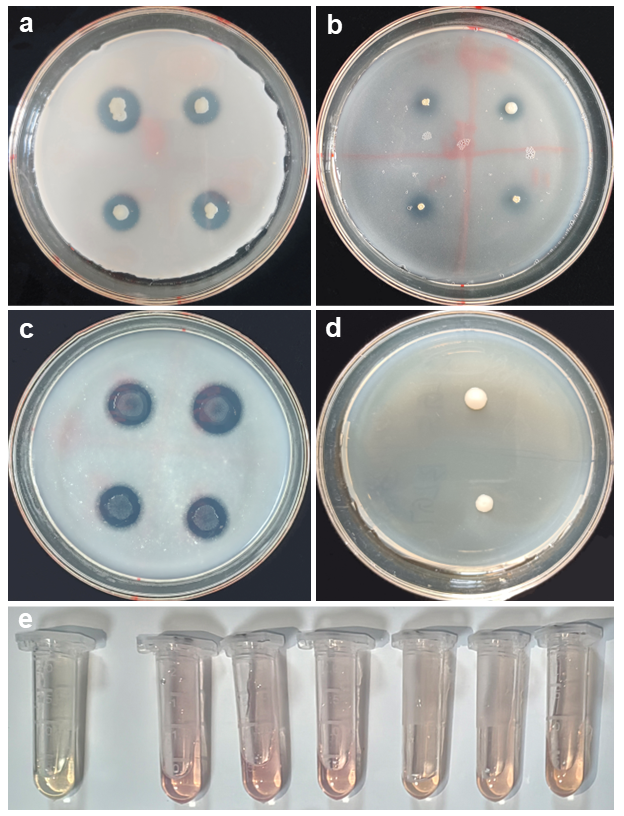


**Supplementary Figure 3** Representative isolates for plant growth promoting traits on (**a**) Organophosphorus Agar Media; (**b**) Mehknha Inorganic Phosphorus Agar Mediua; (**c**) Norris Glucose Nitrogen Free Media; (**d**) ADF-ACC media; (**e**) IAA assay.

**Supplementary Figure 4** IAA standard curve.

[See separate file]

**Supplementary Figure 5** Alpha diversity and Lefse analysis of the rhizosphere bacterial community in *Isatis indigotica*. (**a**) Analysis of alpha diversity of the rhizosphere bacterial community. (**b**, **c**) Analysis of Cladogram (**b**) and LDA score (**c**). Data represent the SDs of three independent experiments. Different lowercase letters indicate a significant difference at *p* < 0.05. The same lowercase letters indicate non-significant differences.

[See separate file]

**Supplementary Figure 6** Alpha diversity and Lefse analysis of the rhizosphere fungi in *Isatis indigotica*. (**a**) Analysis of alpha diversity of the rhizosphere fungi community. (**b**, **c**) Analysis of Cladogram (**b**) and LDA score (**c**). Data represent the SDs of three independent experiments. Different lowercase letters indicate a significant difference at *p* < 0.05. The same lowercase letters indicate non-significant differences.
